# 14-3-3 $\upzeta /\updelta$-reported early synaptic injury in Alzheimer’s disease is independently mediated by sTREM2

**DOI:** 10.1186/s12974-023-02962-z

**Published:** 2023-11-24

**Authors:** Marcel S. Woo, Johanna Nilsson, Joseph Therriault, Nesrine Rahmouni, Ann Brinkmalm, Andrea L. Benedet, Nicholas J. Ashton, Arthur C. Macedo, Stijn Servaes, Yi-Ting Wang, Cécile Tissot, Jaime Fernandez Arias, Seyyed Ali Hosseini, Mira Chamoun, Firoza Z. Lussier, Thomas K. Karikari, Jenna Stevenson, Christina Mayer, João Pedro Ferrari-Souza, Eliane Kobayashi, Gassan Massarweh, Manuel A. Friese, Tharick A. Pascoal, Serge Gauthier, Henrik Zetterberg, Kaj Blennow, Pedro Rosa-Neto

**Affiliations:** 1 Institute of Neuroimmunology and Multiple Sclerosis, University Medical Center Hamburg Eppendorf, Falkenried 94, 20251 Hamburg, Germany; 2 Translational Neuroimaging Laboratory, McGill Research Centre for Studies in Aging, 6875 La Salle Blvd, FBC Room 3149, Montreal, QC H4H 1R3 Canada; 3 Clinical Neurochemistry Laboratory, Sahlgrenska University Hospital, 40530 Gothenburg, Sweden; 4 Department of Neurology and Neurosurgery, Faculty of Medicine, McGill University, Montreal, QC H4H 1R3 Canada; 5 Department of Psychiatry and Neurochemistry, The Sahlgrenska Academy at the University of Gothenburg, 40530 Mölndal, Sweden; 6 Wallenberg Centre for Molecular and Translational Medicine, University of Gothenburg, 40530 Gothenburg, Sweden; 7 Department of Neurology and Psychiatry, University of Pittsburgh School of Medicine, Pittsburgh, PA 15213 PA USA; 8 Graduate Program in Biological Sciences: Biochemistry, Universidade Federal do Rio Grande do Sul, Porto Alegre, RS 91501-970 Brazil; 9 Department of Neurodegenerative Disease, UCL Institute of Neurology, Queen Square, London, WC1E 6BT UK; 10 UK Dementia Research Institute at UCL, London, WC1E 6BT UK; 11 Hong Kong Center for Neurodegenerative Diseases, Clear Water Bay, Hong Kong, 518172 China; 12 Wisconsin Alzheimer’s Disease Research Center, University of Wisconsin School of Medicine and Public Health, University of Wisconsin-Madison, Madison, WI 53726 USA

**Keywords:** Alzheimer’s disease, sTREM2, Microglia, Neuroinflammation, Synaptic loss

## Abstract

**Introduction:**

Synaptic loss is closely associated with tau aggregation and microglia activation in later stages of Alzheimer’s disease (AD). However, synaptic damage happens early in AD at the very early stages of tau accumulation. It remains unclear whether microglia activation independently causes synaptic cleavage before tau aggregation appears.

**Methods:**

We investigated 104 participants across the AD continuum by measuring 14-3-3 zeta/delta ($\upzeta /\updelta$) as a cerebrospinal fluid biomarker for synaptic degradation, and fluid and imaging biomarkers of tau, amyloidosis, astrogliosis, neurodegeneration, and inflammation. We performed correlation analyses in cognitively unimpaired and impaired participants and used structural equation models to estimate the impact of microglia activation on synaptic injury in different disease stages.

**Results:**

14-3-3 $\upzeta /\updelta$ was increased in participants with amyloid pathology at the early stages of tau aggregation before hippocampal volume loss was detectable. 14-3-3 $\upzeta /\updelta$ correlated with amyloidosis and tau load in all participants but only with biomarkers of neurodegeneration and memory deficits in cognitively unimpaired participants. This early synaptic damage was independently mediated by sTREM2. At later disease stages, tau and astrogliosis additionally mediated synaptic loss.

**Conclusions:**

Our results advertise that sTREM2 is mediating synaptic injury at the early stages of tau accumulation, underlining the importance of microglia activation for AD disease propagation.

**Supplementary Information:**

The online version contains supplementary material available at 10.1186/s12974-023-02962-z.

## Introduction

The dominant conceptual framework assumes sequential pathophysiological events leading to neuronal injury and dementia in Alzheimer’s disease (AD) [1]. Neuronal injury is considered a downstream event following the appearance of extracellular amyloid-β aggregates and intra-neuronal neurofibrillary tangles (NFTs) that disrupt functionality and drive neuronal demise [2]. This framework is supported by significant imaging and fluid biomarker literature that reveals early detection of amyloid-β and tau pathology before synaptic loss and neuronal injury can be unequivocally quantified [3, 4]. Synaptic damage measured by FDG-PET and other biomarkers has been considered a marker of neuronal injury [5]. The concept that synaptic damage constitutes a late event in AD pathophysiology is challenged by the strong amyloid-β species neurotoxicity reported in numerous in vitro and in preclinical studies [6–8] as well as biomarker studies that showed increased CSF levels of the neuron-specific protein neurogranin in individuals with amyloid-β pathology in contrast to individuals without amyloid-β accumulation [9]. Moreover, disease-related cognitive decline preceding hippocampal atrophy [10] also supports the concept of early synaptic dysfunction in AD.

Measuring synaptic protein fragments in body fluids constitutes a promising strategy to investigate early synaptic dysfunction in AD [11]. For example, increased CSF levels of the synaptosomal-associated protein 25 kDa (SNAP25) were observed in predementia and dementia phases of AD [12, 13] 14-3-3 $\upzeta$ is a synaptic protein that recently became an attractive biomarker for testing hypotheses related to early synaptic damage in AD. Indeed, it has been demonstrated that CSF levels 14-3-3 $\upzeta /\updelta$ increases along the AD continuum in participants of the Alzheimer’s Disease Neuroimaging Initiative (ADNI) cohort [14].

Activation of the innate immune system co-occurs with tau spreading in AD [15] and several observational studies in humans across the AD continuum revealed increased cytokine levels and other inflammation markers in the CSF of patients with detectable amyloid-β pathology [16]. In accordance, studies in different preclinical AD models demonstrated that pro-inflammatory microglia lead to excessive synaptic loss by phagocytosis [17]. Activated microglia upregulate the Triggering Receptor Expressed in Myeloid Cells 2 (TREM2) which is pattern recognition receptor and can be cleaved to produce a soluble form (sTREM2) [18]. sTREM2 levels in the cerebrospinal fluid (CSF) are associated with tau and amyloid-β load and fluid AD biomarkers [19] and predict the conversion rate of MCI to AD [20]. Furthermore, a recent *post-mortem* study showed that TREM2 expression strongly correlated with core pathologies of AD and cognitive decline [21]. However, microglial activation and upregulation of sTREM2 have been conceptualized as downstream event of tau and amyloid-β accumulations. In its unclear whether sTREM2 contributes to early synaptic cleavage before tau accumulation appears and whether sTREM2 aggravates neuronal loss independently of tau and amyloid-β.

Here, we hypothesized that synaptic damage appears early in the AD continuum when amyloid-β pathology is present at the early stages of NFT accumulation and that the magnitude of 14-3-3 $\upzeta /\updelta$ abnormality correlates with severity of AD pathophysiology. Furthermore, we tested our hypothesis that synaptic damage in early and late AD disease stages are differently mediated by microglial inflammation and astrogliosis. Since TREM2 is strongly upregulated in activated microglia and sTREM2 is increased in the CSF of AD patients, we asked whether sTREM2 mediates early synaptic damage independently of other hallmarks of AD.

## Materials and methods

### Participants

We assessed 20 cognitively unimpaired young (CUY; ages 20–32 years), 44 cognitively unimpaired older participants (CU; ages 40–80 years), 24 participants with mild cognitive impairment (MCI; ages 63–82 years), and 16 AD (ages 53–74 years) participants enrolled in the Translational Biomarkers of Aging and Dementia (TRIAD) cohort [22] who underwent amyloid-β PET with [^18^F]AZD4694, tau-PET with [^18^F]MK6240 and magnetic resonance imaging (MRI). Participants had a detailed clinical and cognitive assessment, including the Clinical Dementia Rating (CDR) and Mini-Mental State Examination (MMSE). CU and CUY participants had no objective cognitive impairment, a CDR score of 0, and were asked to report any subjective cognitive decline in a questionnaire given during screening. Individuals with MCI had subjective cognitive impairment, relatively preserved activities of daily living, and a CDR score of 0.5. Mild-to-moderate Alzheimer’s clinical syndrome patients with dementia had a CDR score between 0.5 and 2 and met the National Institute on Aging—Alzheimer’s Association (NIA-AA) criteria for probable AD determined by a dementia specialist [22]. Exclusion criteria were active substance abuse, recent head trauma, recent major surgery, or MRI/PET safety contraindications [23].

### MRI acquisition and processing

Structural MRI data were acquired at the MNI for all participants on a 3 T Siemens Magnetom scanner using a standard head coil. Hippocampal volume was assessed using FreeSurfer version 6.0 using the Desikan–Killiany–Tourville atlas gray matter segmentation.

### PET acquisition and processing

Study participants had a T1-weighted MRI, and [^18^F]AZD4694 PET, [^18^F]MK6240 PET and microglial activation TSPO [11C]PBR28 PET scans were acquired using a brain-dedicated Siemens high-resolution research tomograph. [^18^F]MK6240 PET images were acquired at 90–110 min after the intravenous bolus injection of the radiotracer and reconstructed using an ordered subset expectation maximization algorithm on a 4D volume with four frames (4 × 300 s), as previously described [24]. [^18^F]AZD4694 PET images were acquired at 40–70 min after the intravenous bolus injection of the radiotracer and reconstructed with the same ordered subset expectation maximization algorithm on a 4D volume with three frames (3 × 600 s) [22]. [^11^C]PBR28 images were acquired at 60–90 min after the intravenous bolus injection of the tracer (mean injected dose = 384 megabecquerel, mean molar activity = 193 gigabecquerel μmol^−1^) and reconstructed using the OSEM algorithm on a 4D volume with 6 frames (6 × 300 s) [15]. A 6-min transmission scan with a rotating ^137^Cs point source was conducted at the end of each PET acquisition for attenuation correction. Images were corrected for motion, decay, dead time and random and scattered coincidences. In summary, PET images were linearly registered to T1-weighted image space, and the T1-weighted images were linearly and nonlinearly registered to the Alzheimer’s Disease Neuroimaging Initiative (ADNI) reference space. To minimize the influence of meningeal spillover into adjacent brain regions, [^18^F]MK6240 images were skull-striped in T1 space before transformations and blurring [23]. The PET images in T1-space were linearly and nonlinearly registered to the ADNI space using transformations from the T1-weighted image to ADNI space. [^18^F]MK6240 SUVRs were calculated using the cerebellar crus I gray matter as a reference region [23], as derived from the SUIT cerebellum atlas [25]. [^18^F]AZD4694 SUVRs were calculated using the whole cerebellum gray matter as the reference region. PET images were spatially smoothed to achieve an 8-mm full-width at half-maximum resolution. The global [^18^F]AZD4694 SUVR composite included the precuneus, prefrontal, orbitofrontal, parietal, temporal and cingulate cortices [25]. Participants were assigned according to the A/T/N framework by measuring whole brain [^18^F]MK6240 SUVR (cut-off 1.24), neocortical [^18^F]AZD4694 SUVR (cut-off 1.55) and hippocampal volume by structural MRI (cut-off 3.2 cm^3^) as previously described [22, 24].

### PET-based Braak staging methods

The transentorhinal cortex was segmented in the stereotaxic space on 1-mm isotropic voxels [23] using a validated MRI segmentation technique and identifiable anatomical landmarks [26]. The transentorhinal ROI was segmented in the medial bank of the rhinal sulcus, which corresponds to the transition area between the entorhinal and perirhinal cortices [27]. The transentorhinal and entorhinal cortex regions of interest (ROIs) did not overlap with each other. The peaks of the transentorhinal and entorhinal cortex ROI probabilistic distributions were 14 mm apart, which is just under two full widths at half-maximum of the PET resolution in this study (8 mm). PET Braak-like stage segmentation was previously described [15, 23]. Stages included the following regions: Braak I (transentorhinal), Braak II (entorhinal and hippocampus), Braak III (amygdala, para-hippocampal gyrus, fusiform gyrus and lingual gyrus), Braak IV (insula, inferior temporal, lateral temporal, posterior cingulate and inferior parietal), Braak V (orbitofrontal, superior temporal, inferior frontal, cuneus, anterior cingulate, supramarginal gyrus, lateral occipital, precuneus, superior parietal, superior frontal and rostromedial frontal) and Braak VI (paracentral, postcentral, precentral and pericalcarine) [28, 29].

### Fluid biomarkers

CSF samples were collected with lumbar puncture under local anesthesia. 29 mL fluid was collected with polypropylene syringes, from which the initial 4 mL was sent to local laboratory for routine analyses. The remaining volume was transferred to polypropylene tubes and centrifuged at 20 °C for 10 min at 2200 g. Samples were then rapidly frozen for permanent storage at − 80 °C. CSF concentrations of amyloid-β (Aβ40 and Aβ42) and total Tau were assessed using the fully automated LUMIPULSE G1200 instrument (Fujirebio). Plasma levels of phosphorylated tau (pTau) variants pTau-181, pTau-217, and neurofilament light chain (NfL) and plasma levels of glial fibrillary acidic protein (GFAP) were quantified using a custom Single molecule array (Simoa) assay as previously described [30]. Synaptic biomarkers in the CSF were quantified using mass spectrometry as previously described [31]. In short, a mixture of stable-isotope-labeled peptides as internal standard was added (25 µL, 0.032 pmol/µL) to 100 µL of CSF. Samples were further reduced, alkylated, and tryptic digested and afterward solid-phase extraction was performed for purification. Quantification was executed on a micro-high-performance liquid chromatography–mass spectrometry system (6495 Triple Quadrupole LC/MS system, Agilent Technologies) equipped with a Hypersil Gold reversed-phase C18 column (dim. = 100 × 2.1 mm, particle size = 1.9 µm, Thermo Fisher Scientific), for detailed settings see Additional file 1: Table S1. LC–MS data were analyzed using the software Skyline v. 21 (MacCoss Lab, University of Washington). CSF sTREM2 concentration was measured using a Meso-Scale Discovery assay, as described previously [18].

### Statistical analysis

All statistical analyses were performed in the R software (version 4.0.3). Comparisons of 14-3-3 $\upzeta /\updelta$ between different groups were performed using generalized linear models with adjustment for sex, age and APOE ε4 status. For correlation analysis, mean scaling was performed for 14-3-3 $\upzeta /\updelta$. Correlation analyses were performed using Spearman correlation. False discovery rate (FDR)-adjustment was performed for multiple comparisons. The area under the curve (AUC) for fluid biomarkers was determined using the *pROC* package. Comparisons of AUCs were performed using the Venkatraman test with 2000 permutations. Structural equation modeling was performed to calculate direct and indirect effects was performed using the *lavaan* package [32]. A bootstrap method repeated 5000 times tested the statistical significance of the model’s Chi-square and parameter estimates. The fit of the structural equation models was classified as good if: root mean squared error of approximation < 0.08, comparative fit index > 0.97, and standardized root mean square residual < 0.05, *P*-value of Chi-square estimation > 0.05. z-scoring was performed with all parameters before entering SEMs. SEMs were calculated in A + T − N − and across the whole AD continuum. sTREM2 and GFAP were used as independent variables while 14-3-3 $\upzeta /\updelta$ was entered as the dependent variable. [^18^F]MK-6240 SUVR and [^18^F]AZD-4694 SUVR were treated as mediators. Effects were controlled for age, sex, and educational years. *P*-values < 0.05 were considered statistically significant.

### Ethical approval

The study was approved by the MNI PET working committee and the Douglas Mental Health University Institute Research Ethics Board. Written informed consent was obtained for all participants.

## Results

### Patient demographics

We measured 14-3-3 $\upzeta /\updelta$ levels in the CSF of 104 participants. We included 20 cognitively unimpaired young (CUY; mean/SD age 22.8/0.6 years), 44 cognitively unimpaired older participants (CU; mean/SD age 69/9.2 years), 24 MCI participants (mean/SD age 71.6/8.9 years), and 16 AD participants (mean/SD age 63.9/9.3 years); in total 68 patients were female (59.7%). The patients’ demographics are summarized in Table 1.

**Table 1:** Patient characteristics

Diagnosis	*N* (% female)	Age, mean (SD)	MMSE, mean (SD)	Educational years, mean (SD)	APOE ε4 carriers, *N* (%), N carriership on 1/2 alleles
CUY	20 (70)	23.1 (2.8)	29.8 (0.4)	17.1 (2.6)	6 (30), 6/0
CU	44 (59.1)	69.3 (9.4)	29.1 (0.9)	15.1 (3.4)	13 (29.5), 12/1
MCI	24 (58.3)	71.3 (6)	27.9 (1.6)	15.2 (3.1)	11 (45.8), 11/0
AD	16 (43.8)	63.9 (6.5)	20.8 (6.1)	15.2 (3)	11 (68.8), 8/3

CUY = cognitively normal young, CU = cognitively normal, MCI = mild cognitive impairment, AD = Alzheimer’s disease

### 14-3-3 $\zeta /\delta$ is a biomarker for early synaptic damage

**Fig. 1:**
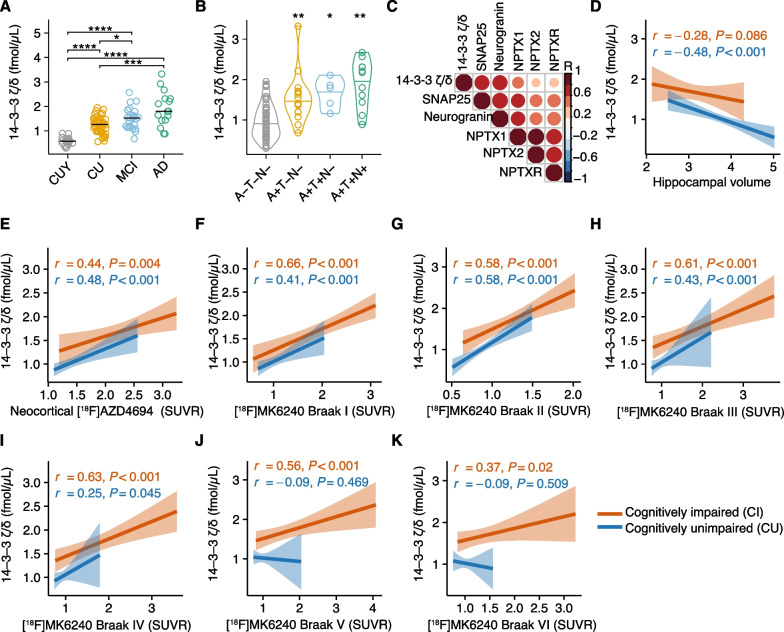
14-3-3 $\upzeta /\updelta$ is upregulated in early disease stages.** A** 14-3-3 $\upzeta /\updelta$ CSF levels in CUY, CU, MCI, and AD. Wilcoxon test with FDR-correction for multiple comparisons was used for statistical comparisons. *P < 0.05, **P < 0.01, ***P < 0.001, ****P < 0.0001. **B** 14-3-3 $\upzeta /\updelta$ CSF levels in A − T − N −, A + T − N −, A + T + N −, and A + T + N + according to the A/T/N framework using [^18^F]AZD4694, [^18^F]MK6240 and hippocampal volume. Wilcoxon test against A − T − N − with FDR-correction for multiple comparisons was conducted. *P < 0.05, **P < 0.01. **C** Correlation of matrix of depicted synaptic proteins in the CSF of all included participants. Color represents correlation coefficient; all correlations were significant with a *P* < 0.05. Spearman correlation was used. **D–K** Spearman correlation analysis of CSF levels of 14-3-3 $\upzeta /\updelta$ with hippocampal volume (**D**), cortical [^18^F]AZD4694 SUVR (**E**), Braak I [^18^F]MK6240 SUVR (**F**), Braak II [^18^F]MK6240 SUVR (**G**), Braak III [^18^F]MK6240 (**H**), Braak IV [^18^F]MK6240 SUVR (**I**), Braak V [^18^F]MK6240 (**J**), Braak VI [^18^F]MK6240 SUVR (**K**) in cognitively impaired and unimpaired participants. *P*-values and correlation coefficients are shown in the figure. CUY = cognitively unimpaired young, CU = cognitively unimpaired, MCI = mild cognitive impairment, AD = Alzheimer’s disease, SNAP25 = synaptosomal-associated protein of 25 kDa, NPTX1 = neuronal pentraxin 1, NPTX2 = neuronal pentraxin 2, NPTXR = neuronal pentraxin receptor

We first compared the 14-3-3 $\upzeta /\updelta$ levels between CUY, CU older individuals, MCI, and AD participants (Fig. 1A). We found that 14-3-3 $\upzeta /\updelta$ was increased age-dependently in CU participants and in MCI and AD participants in comparison to all other groups. Next, we divided our cohort according to the A/T/N framework and compared the 14-3-3 $\upzeta /\updelta$ levels between A − T − N −, A + T − N −, A + T + N − and A + T + N + (Fig. 1B). Intriguingly, 14-3-3 $\upzeta /\updelta$ was already significantly increased in participants who displayed only an amyloid-β positivity without positivity for tau or neurodegenerative biomarkers (A + T − N −). Notably, we still observed this effect after excluding CUY participants for the analysis (Additional file 1: Fig. S1A). Participants with additional tau aggregation and hippocampal volume did not have further increased 14-3-3 $\upzeta /\updelta$ CSF levels underlining that 14-3-3 $\upzeta /\updelta$-reported synaptic damage appears before tau accumulation is visible. Of note, 14-3-3 $\upzeta /\updelta$ was not higher in patients with APOE ε4 carriership on both alleles in comparison to carriership on one allele or without carriership in CI and CU (Additional file 1: Fig. S1B-C). Furthermore, 14-3-3 $\upzeta /\updelta$ significantly correlated with CSF levels of other biomarkers of synaptic injuries such as SNAP25, neuronal pentraxin 1, neuronal pentraxin 2, neuronal pentraxin receptor and neurogranin (Fig. 1C), highlighting that increased 14-3-3 $\upzeta /\updelta$ levels reflect synaptic injury. We concluded that 14-3-3 $\upzeta /\updelta$ is increased in the CSF early in the disease course and before hippocampal atrophy can be quantified by MRI.

After demonstrating that CSF levels of 14-3-3 $\upzeta /\updelta$ are increased in early disease stages, we analyzed its association with other hallmarks of AD in CU and CI participants. First, we performed correlation analysis with the hippocampal volume and found a significant negative correlation in CU but not CI participants (Fig. 1D). Next, we correlated the 14-3-3 $\upzeta /\updelta$ levels with cortical amyloid-β and tau tangle levels across different Braak-like regions measured by PET imaging. We found that 14-3-3 $\upzeta /\updelta$ significantly correlated with amyloid-β deposition in CI and CU (Fig. 1E) and tau tangle levels across Braak-like regions I–IV in CI and CU (Fig. 1F–I). In Braak-like regions V and VI 14-3-3 $\upzeta /\updelta$ only positively correlated with tau tangle load in CI participants (Fig. 1J, K).

**Fig. 2:**
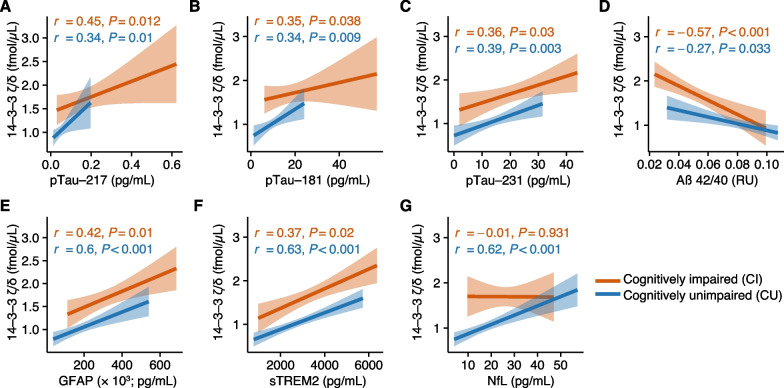
14-3-3 $\upzeta /\updelta$ correlates with hallmarks of AD pathophysiology.** A–G** Spearman correlation of analysis of 14-3-3 $\upzeta /\updelta$ with plasma pTau-217 (**A**), plasma pTau-181 (**B**), plasma pTau-231 (**C**), CSF Aβ42/40 (**D**), plasma GFAP (**E**), CSF sTREM2 (**F**), and plasma NfL (**G**) in cognitively impaired and unimpaired participants. Exact *P*-values and correlation coefficients are shown in the figure

Additionally, we investigated the associations of 14-3-3 $\upzeta /\updelta$ with other CSF and plasma biomarkers that reflect amyloidosis, tau phosphorylation, neurodegeneration, and astrogliosis/glial activation. 14-3-3 $\upzeta /\updelta$ significantly correlated with phosphorylated tau isoforms measured in plasma (Fig. 2A, pTau-217; Fig. 2B, pTau-181; Fig. 2C, pTau-231) and was significantly associated with a decreased CSF Aβ 42/40 ratio (Fig. 2D), increased plasma levels of the astrogliosis biomarker glial fibrillary acid protein (GFAP; Fig. 2E), and increased CSF levels of the microglial activation marker sTREM2 (Fig. 2F) in CI and CU participants. In contrast, CSF 14-3-3 $\upzeta /\updelta$ was only significantly associated with plasma levels of neurofilament light chain (NfL) in CU but not in CI (Fig. 2G). Since the two biomarkers of neurodegeneration hippocampal volume and plasma NfL were only significantly associated with CSF 14-3-3 $\upzeta /\updelta$ in CU, we next tested whether this is clinically reflected in the participants. Indeed, we found that CSF 14-3-3 $\upzeta /\updelta$ was significantly correlated with memory deficits only in CU but not in CI (Additional file 1: Fig. S1D-E) supporting that CSF levels 14-3-3 $\upzeta /\updelta$ reflect early synaptic dysfunction.

### 14-3-3 $\zeta /\delta$ identifies brain amyloid pathology

**Fig. 3:**
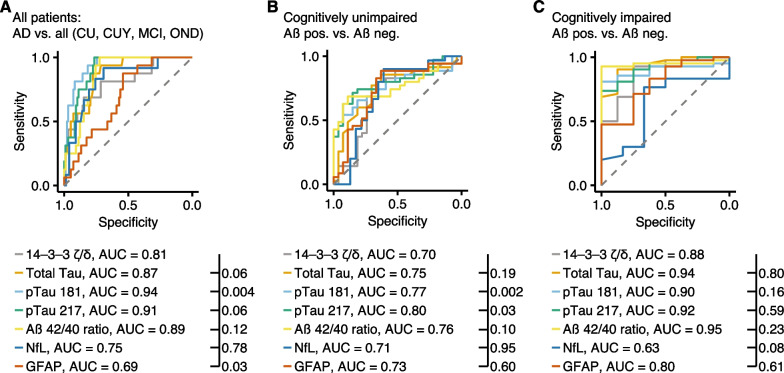
14-3-3 $\upzeta /\updelta$ discriminates carriers of amyloid β.** A–C** ROC analyses contrasting CSF biomarkers between AD and all other groups (**A**), amyloid-β positive and amyloid-β negative cognitively unimpaired (**B**) and amyloid β positive and amyloid β negative cognitively impaired (**C**) individuals. AUCs and *P*-values of statistical comparisons between 14-3-3 $\upzeta /\updelta$ and all other biomarkers are shown in the figure. ROC curves were compared using Venkatraman test

Next, we investigated the predictive performance of CSF *14-3-3* $\upzeta /\updelta$ in comparison with other known biomarkers to identify amyloid-β positivity in cognitively unimpaired and impaired participants. First, we compared the AUCs of AD against all other groups (CU, CUY, MCI) and compared 14-3-3 $\upzeta /\updelta$ with other known biomarkers of AD (Fig. 3A). The AUC of 14-3-3 $\upzeta /\updelta$ (AUC = 0.81) was significantly increased in comparison to GFAP (AUC = 0.69, *P* = 0.03) but decreased as compared to pTau-181 (AUC = 0.94, *P* = 0.004), but not pTau-217 (AUC = 0.91, *P* = 0.06) and total Tau (AUC = 0.87, *P* = 0.06). No differences were found in comparison to Aβ 42/40 (AUC = 0.89, *P* = 0.12). Next, we separated (CU) and cognitively impaired (CI) participants (namely, MCI and AD pooled) with and without brain amyloidosis as measured by PET. In CU (Fig. 3B) we found that 14-3-3 $\upzeta /\updelta$ showed decreased AUC (AUC = 0.70) in comparison to pTau-181 (AUC = 0.77, *P* = 0.002) and pTau-217 (AUC = 0.8, *P* = 0.03) but, again, no difference when compared to Aβ 42/40 (AUC = 0.76, *P* = 0.1). In contrast, in CI individuals (Fig. 3C), the AUC of 14-3-3 $\upzeta /\updelta$ to differentiate amyloid-β pathology (AUC = 0.88) was comparable to those of pTau-181 (AUC = 0.90, *P* = 0.16), pTau-217 (AUC = 0.92, *P* = 0.59) and Aβ 42/40 (AUC = 0.95, *P* = 0.23) underlining its sensitivity to report amyloid-β brain load in early and late AD pathophysiology.

### sTREM2 independently mediates synaptic damage in early and late AD

**Fig. 4:**
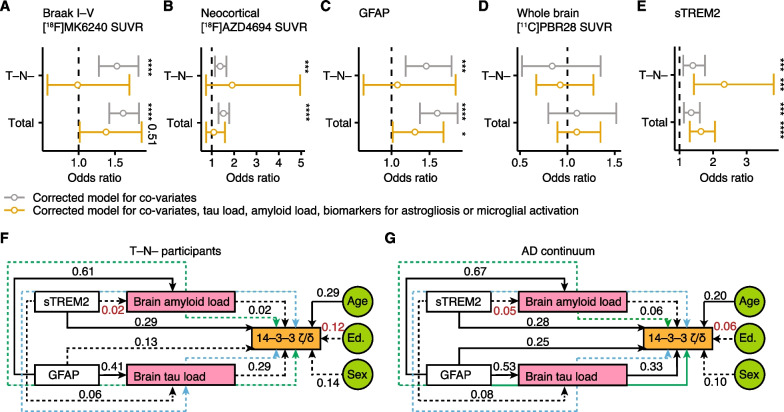
sTREM2 mediates early synaptic loss. **A–E** Odds ratio (OR) analysis of [18F]MK6240 SUVR Braak I-V (**A**), neocortical [^18^F]AZD4694 SUVR (**B**), plasma GFAP (**C**), whole brain [^11^C]PBR28 SUVR (**D**) and CSF sTREM2 (**E**) in T-N- participants and across the whole continuum. ORs, 5% and 95% confidence intervals are displayed corrected for covariates (sex, age, educational years, *APOE* ε4 status) or covariates and respective other biomarkers for tau load, amyloid load, astrogliosis and microglial activation.** F** and** G** Structural equation models of CSF sTREM2 and plasma GFAP with 14-3-3 $\upzeta /\updelta$ in T-N-participants (**F**) and across the AD continuum (**G**). [18F]MK6240 SUVR Braak I–V and [^18^F]AZD4694 SUVR were tested as mediators. Goodness-of-fit indicators are provided in Additional file 1: **Table S3**. Black lines show direct effects, green lines show GFAP-mediated effects, blue lines show sTREM2-mediated effects, solid lines show significant and dashed lines show not significant effects. Standardized estimates are provided in the figure. *P < 0.05, **P < 0.01, ***P < 0.001, ****P < 0.0001

Next, we aimed to find mediators of increased 14-3-3 $\upzeta /\updelta$ CSF levels in different pathophysiological disease stages. Therefore, we separately analyzed participants without brain tau accumulation and hippocampal volume loss (T-N-) and all participants across the whole AD continuum and calculated odds ratios of brain tau load (Fig. 4A), brain amyloid load (Fig. 4B), plasma GFAP (Fig. 4C), general microglial load measured by PET (Fig. 4D) and CSF sTREM2 (Fig. 4E) to predict the magnitude of 14-3-3 $\upzeta /\updelta$ in the CSF. Of note, GFAP (Additional file 1: Fig. S2A) and total [11C]PBR28 (Additional file 1: Fig. S2B) were significantly increased in A + T-N- in comparison to A-T-N- which was not the case for sTREM2 (Additional file 1: Fig. S2C). After correction for covariates (age, sex, APOE ε4 status), brain tau and amyloid accumulation, astrogliosis and increased sTREM2 levels were associated with 14-3-3 $\upzeta /\updelta$ in T-N- and across the whole AD continuum. However, when correcting for covariates and respective other AD hallmarks only sTREM2 (*P* < 0.001) and GFAP (*P* = 0.04) significantly predicted increased 14-3-3 $\upzeta /\updelta$ levels across the AD continuum. Intriguingly, in T-N- only sTREM2 (*P* = 0.007) was significantly associated with synaptic loss after correcting for covariates, brain amyloid and tau load, astrogliosis and microglia load underlining that sTREM2 independently mediates synaptic damage before tau accumulation occurs.

After identifying astrogliosis and sTREM2 as major predictors of increased 14-3-3 $\upzeta /\updelta$ in the CSF we analyzed, whether these independently mediate synaptic release of 14-3-3 $\upzeta /\updelta$ and tested [^18^F]MK6240 and [^18^F]AZD4694 as possible mediators. Therefore, we separately performed structural equation models for T-N- participants and all participants (goodness-of-fit indices are provided in Additional file 1: Table S2). In accordance with our odds ratio analyses, in T-N- only sTREM2 significantly mediated synaptic loss but not GFAP, and tau load (Fig. 4F). Notably, we only observed a significant direct mediation by sTREM2 (standardized estimate (st. est.) = 0.29, *P* = 0.02) but not indirect mediations through brain amyloidosis and tau accumulation supporting that sTREM2 independently induces synaptic loss in early disease stages (direct and indirect effects are summarized in Table 2). When looking across the whole AD continuum (Fig. 4G) GFAP (st. est. = 0.25, *P* = 0.02) and brain tau load (st. est. = 0.33, *P* = 0.006) in addition to sTREM2 (st. est. = 0.28, *P* = 0.001) were significantly mediating synaptic damage. Again, sTREM2 was not significantly associated with brain tau load and we did not observe significant indirect mediation effects. In contrast, GFAP was strongly associated with brain amyloidosis (st. est. = 0.67, *P* < 0.001) and tau accumulation (st. est. = 0.53, *P* < 0.001) and we identified brain tau load as significant indirect mediator (st. est. = 0.18, *P* = 0.03) of GFAP-associated synaptic damage (direct and indirect effects are summarized in Table 3).

**Table 2:** Structural equation model of sTREM2-mediated early synaptic damage

Effectors	Mediators	Effect	SE	z-value	*P*-value	St. estimate
sTREM2 + GFAP	[18F]MK6240 + [18F]AZD4694	Combined	0.15	3.43	< 0.001	0.57
GFAP	[18F]AZD4694	Indirect	0.09	0.3	0.76	0.03
sTREM2	[18F]AZD4694	Indirect	0.01	-0.08	0.94	− 0.001
GFAP	[18F]MK6240	Indirect	0.07	1.42	0.15	0.11
sTREM2	[18F]MK6240	Indirect	0.03	0.59	0.56	0.02

SE = standard error, st. estimate = standardized estimate, GFAP = glial fibrillary acid protein, sTREM2 = soluble triggering receptor expressed in myeloid cells 2 (sTREM2)

**Table 3:** Structural equation model of sTREM2 and GFAP-induced synaptic damage

Effectors	Mediators	Effect	SE	z-value	*P*-value	St. estimate
sTREM2 + GFAP	[18F]MK6240 + [18F]AZD4694	Combined	0.15	5.35	< 0.001	0.78
GFAP	[18F]AZD4694	Indirect	0.08	0.64	0.52	0.05
sTREM2	[18F]AZD4694	Indirect	0.01	-0.24	0.81	− 0.003
GFAP	[18F]MK6240	Indirect	0.08	2.2	0.03	0.17
sTREM2	[18F]MK6240	Indirect	0.04	0.67	0.5	0.03

SE = standard error, st. estimate = standardized estimate, GFAP = glial fibrillary acid protein, sTREM2 = soluble triggering receptor expressed in myeloid cells 2 (sTREM2)

## Discussion

This study evaluated synaptic damage across the spectrum of clinical presentation of AD by measuring 14-3-3 $\upzeta /\updelta ,$ a biomarker [14, 33] of synaptic injury. Thus far, neuronal injury quantified by Neurofilament light chain (NfL) or hippocampal atrophy has been considered a late event in AD pathology which appears years after amyloid and tau load [34–36]. However, we found that 14-3-3 $\upzeta /\updelta$ was already upregulated in participants who only displayed amyloid-β accumulation in the CNS before tau aggregation or hippocampal atrophy appeared. This is in line with other studies that demonstrated increased levels of other markers of synaptic dysfunction and degeneration such as neurogranin or SNAP-25 in MCI and AD dementia patients [11, 37]. Furthermore, 14-3-3 $\upzeta /\updelta$ was significantly associated with hippocampal volume loss, NfL and memory deficits in CU but not CI patients. Thus, our data support a conceptual framework in which neuronal injury and synaptic dysfunction are early events in AD pathophysiology that happen independent of tau accumulation.

Next, we set out to find mediators of synaptic damage across the AD continuum and tested imaging and fluid biomarkers of astrogliosis, microglia, brain amyloidosis and tau accumulation. In participants who did not display brain tau load or hippocampal atrophy we identified sTREM2 as decisive mediator of synaptic injury. Our findings are supported by a recent study that identified sTREM2 as important mediator of amyloid-β induced tau phosphorylation in early amyloid-β accumulators. Furthermore, an early increase of sTREM2 is predictive for conversion of MCI to AD [20] and mechanistic findings revealed sTREM2 as an important inductor of inflammatory microglia and synaptic phagocytosis [38]. A recent preclinical study demonstrated in an amyloid-β dependent AD mouse model that damaged synapses are engulfed by sTREM2 and are removed by microglia [39] reducing pathological cortical hyperexcitability. TREM2 is upregulated in microglia which are in proximity to extracellular plaques [40, 41] and TREM2 activating antibodies enhance amyloid-β clearance in AD mouse models [42, 43]. On the other hand, early synaptic loss through inflammatory microglia support disease progression and neurodegeneration in preclinical studies [17, 44] underlining the complexity of microglia for AD pathophysiology. Our data further support the importance for sTREM2-mediated synaptic stripping. However, it remains unclear whether the findings from mouse studies can be translated into human AD and the observed sTREM2-mediated increase of 14-3-3 $\upzeta /\updelta$ in the CSF reflects clearance of damaged synapses or another type of synaptic injury.

In later disease stages when tau accumulation was visible, we additionally identified GFAP [45, 46] as significant mediator of 14-3-3 $\upzeta /\updelta$ in the CSF which is in line with preclinical studies that identified complement-dependent synaptic phagocytosis by astrocytes and microglia [44]. Intriguingly, SEM analysis revealed that brain tau load acted as significant mediator of astrogliosis induced increase of 14-3-3 $\upzeta /\updelta$. Interestingly, recent studies showed that GFAP-reported astrogliosis is mainly driven by brain amyloidosis and not tau accumulation [47]. Together our data support that along the sequential AD pathophysiology reactive astrocytes might be induced by extracellular amyloid-β plaques and subsequently support spreading of NFTs and neurodegeneration as demonstrated in neuronal cell cultures [48].

Limitations of our study include the cross-sectional design. Longitudinal follow-up with amyloid-β reducing treatments is required to investigate the interaction between sTREM2 and AD pathology. In addition, this is a monocentric study with a well-characterized memory clinic cohort. Further validation in independent cohorts is required. Although our findings are strengthened by employing human data additional functional mouse and cell culture studies are needed to establish the causative relationships between sTREM2, and synaptic release of 14-3-3 $\upzeta /\updelta$. Especially, the relationship between different biomarkers of synaptic injury and neuronal pathologies should be the focus of prospective mouse studies to help interpretation of the synaptic biomarkers. Additionally, comparing SV2A PET [49] with CSF levels of 14-3-3 $\upzeta /\updelta$ across the AD continuum would strengthen its use as fluid biomarker to measure synaptic injury in AD.

In summary, our results support that synaptic injury begins early in AD pathophysiology at the early stages of tau aggregation and before neuronal loss with hippocampal atrophy are visible. Mechanistically, our data support that early synaptic injury is independently mediated by sTREM2, whereas later synaptic damage is additionally driven by astrogliosis and tau accumulation. Therefore, our data advocate the importance of early detection of amyloid-β pathology to prevent synaptic damage and underlines that sTREM2 is a key player in early AD disease stages.

### Supplementary Information

**Additional file 1: Table S1. **LC–MS settings. LC–MS settings including electrospray and iFunnel for the analysis of the synaptic protein panel. **Table S2. **Odds ratio analyses to predict 14-3-3 $\upzeta /\updelta$. OR = Odds ratio, CI = Confidence interval (up = 95%, low = 5%), GFAP = glial fibrillary acid protein, sTREM2 = soluble triggering receptor expressed in myeloid cells 2 (sTREM2). **Table S3. **SEM indices of goodness of fit. CFI = Comparative Fit Index, RMSEA = Root Mean Square Error of Approximation, SRMR = Standardized Root Mean Square Residual, TLI = Tucker Lewis Index, AD = Alzheimer’s Disease. **Figure S1. **14-3-3 $\upzeta /\updelta$ is associated with memory deficits in cognitively unimpaired. (A) CSF levels of 14-3-3 $\upzeta /\updelta$ in A-T-N-, A + T-N-, A + T + N-, and A + T + N + , cognitively unimpaired young individuals were excluded for this analysis. Wilcoxon test with FDR-correction for multiple comparisons was used for statistical comparisons. * *P* < 0.05, *** P < 0.001. (B-C) CSF levels of 14-3-3 $\upzeta /\updelta$ in CU (B) and CI (C) participants divided by APOE ε4 carriership. (D-E) Spearman correlation analyses between CSF levels of 14-3-3 $\upzeta /\updelta$ and RAVLT immediate score (D) and delayed score (E) in cognitively unimpaired and impaired participants. Exact *P*-values and correlation coefficients are shown in the figure. **Figure S2. **Astrogliosis and microglia activation are detectable in early disease. (A–C) Plasma GFAP levels (A), whole brain [^11^C]PBR28 SUVR (B) and CSF sTREM2 (C) levels in A–T–N–, A + T–N–, A + T + N–, and A + T + N + according to the A/T/N framework using [^18^F]AZD4694, [^18^F]MK6240 and hippocampal volume. Wilcoxon test against A–T–N– with FDR-correction for multiple comparisons were conducted. *P < 0.05, **P < 0.01, ***P < 0.001.

## Data Availability

All the data supporting the findings of this study are available on reasonable request from the corresponding author.

## Abbreviations

AD: Alzheimer’s disease
CI: Confidence interval
CU: Cognitively unimpaired
CUY: Cognitively unimpaired young
CSF: Cerebrospinal fluid
GFAP: Glial fibrillary acid protein
MCI: Mild cognitive impairment
MMSE: Mini-Mental-State-Examination
NFT: Neurofibrillary tangles
SD: Standard deviation
SEM: Structural equation model
OND: Other neurodegenerative disease
RAVLT: Rey’s Auditory Verbal Learning Test
sTREM2: Soluble triggering receptor expressed in myeloid cells
SUVR: Standardized uptake value ratio
