# Supplementary material for: 14-3-3 ζ/δ-reported early synaptic injury in Alzheimer’s disease is independently mediated by sTREM2
Source: J Neuroinflammation. 2023 Nov 24;20:278. doi: 10.1186/s12974-023-02962-z (PMC10675887; doi:10.1186/s12974-023-02962-z)
Supplement: Supplementary file 1 — Additional file 1: Table S1. LC–MS settings. LC–MS settings including electrospray and iFunnel for the analysis of the synaptic protein panel. Table S2. Odds ratio analyses to predict 14-3-3 $\upzeta /\updelta$ζ/δ. OR = Odds ratio, CI = Confidence interval (up = 95%, low = 5%), GFAP = glial fibrillary acid protein, sTREM2 = soluble triggering receptor expressed in myeloid cells 2 (sTREM2). Table S3. SEM indices of goodness of fit. CFI = Comparative Fit Index, RMSEA = Root Mean Square Error of Approximation, SRMR = Standardized Root Mean Square Residual, TLI = Tucker Lewis Index, AD = Alzheimer’s Disease. Figure S1. 14-3-3 $\upzeta /\updelta$ζ/δ is associated with memory deficits in cognitively unimpaired. (A) CSF levels of 14-3-3 $\upzeta /\updelta$ζ/δ in A-T-N-, A + T-N-, A + T + N-, and A + T + N + , cognitively unimpaired young individuals were excluded for this analysis. Wilcoxon test with FDR-correction for multiple comparisons was used for statistical comparisons. * P < 0.05, *** P < 0.001. (B-C) CSF levels of 14-3-3 [file 12974_2023_2962_MOESM1_ESM.docx]

|  | **Parameter** | **Setting** |
| --- | --- | --- |
| **LC** | Sample injection volume | 40 µL |
|  | Flow-rate | 0.3 mL/min |
|  | Gradient | Broken; 5–20%B (20 min), 20–35%B (7 min) |
|  | Total cycle time | 30 min |
|  | Mobile phase A | 0.1% formic acid in water (v/v) |
|  | Mobile phase B | 0.1% formic acid/84% acetonitrile in water (v/v) |
| **Electrospray** | Gas temperature | 220 °C |
|  | Gas flow | 15 L/min |
|  | Nebulizer pressure | 40 psi |
|  | Sheath gas temperature | 200 °C |
|  | Sheath gas flow | 11 L/min |
|  | Capillary voltage | 3500 V |
|  | Nozzle voltage | 500 V |
| **iFunnel** | High-pressure radio frequency | 200 V |
|  | Low-pressure radio frequency | 160 V |
| **MRM** | Retention time windows | 0.8 min |
|  | Collision energies | Individually optimized |
|  | Cell accelerator voltages | Individually optimized |

**Table S1.** LC-MS settings including electrospray and iFunnel for the analysis of the synaptic protein panel.

| Predictor | Correction | Participants | OR | CI low | CI up | P value |
| --- | --- | --- | --- | --- | --- | --- |
| GFAP | Covariates | AD continuum | 1.603 | 1.373 | 1.871 | < 0.001 |
| sTREM2 | Covariates | AD continuum | 1.347 | 1.131 | 1.605 | 0.001 |
| [11C]PBR28 | Covariates | AD continuum | 1.1 | 0.798 | 1.516 | 0.565 |
| [18F]MK6240 | Covariates | AD continuum | 1.611 | 1.42 | 1.828 | < 0.001 |
| [18F]AZD4694 | Covariates | AD continuum | 1.517 | 1.299 | 1.772 | < 0.001 |
| GFAP | Covariates+respective other biomarkers | AD continuum | 1.308 | 1.016 | 1.684 | 0.048 |
| sTREM2 | Covariates+respective other biomarkers | AD continuum | 1.638 | 1.306 | 2.055 | < 0.001 |
| [11C]PBR28 | Covariates+respective other biomarkers | AD continuum | 1.099 | 0.894 | 1.349 | 0.379 |
| [18F]MK6240 | Covariates+respective other biomarkers | AD continuum | 1.376 | 1.015 | 1.865 | 0.051 |
| [18F]AZD4694 | Covariates+respective other biomarkers | AD continuum | 1.087 | 0.745 | 1.584 | 0.67 |
| GFAP | Covariates | T–N– | 1.458 | 1.187 | 1.791 | 0.001 |
| sTREM2 | Covariates | T–N– | 1.397 | 1.111 | 1.756 | 0.006 |
| [11C]PBR28 | Covariates | T–N– | 0.839 | 0.521 | 1.351 | 0.481 |
| [18F]MK6240 | Covariates | T–N– | 1.525 | 1.278 | 1.821 | < 0.001 |
| [18F]AZD4694 | Covariates | T–N– | 1.373 | 1.14 | 1.654 | 0.001 |
| GFAP | Covariates+respective other biomarkers | T–N– | 1.079 | 0.632 | 1.844 | 0.786 |
| sTREM2 | Covariates+respective other biomarkers | T–N– | 2.333 | 1.429 | 3.807 | 0.007 |
| [11C]PBR28 | Covariates+respective other biomarkers | T–N– | 0.924 | 0.67 | 1.274 | 0.639 |
| [18F]MK6240 | Covariates+respective other biomarkers | T–N– | 0.985 | 0.572 | 1.698 | 0.958 |
| [18F]AZD4694 | Covariates+respective other biomarkers | T–N– | 1.909 | 0.735 | 4.955 | 0.214 |

**Table S2. Odds ratio analyses to predict 14-3-3** $\boldsymbol{\zeta/\delta}$**.** OR = Odds ratio, CI = Confidence interval (up = 95%, low = 5%), GFAP = glial fibrillary acid protein, sTREM2 = soluble triggering receptor expressed in myeloid cells 2 (sTREM2).

| Parameters | T–N– | AD continuum |
| --- | --- | --- |
| Chi-square P-value | 0.33 | 0.07 |
| CFI | 0.99 | 0.98 |
| RMSEA | 0.05 | 0.07 |
| SRMR | 0.04 | 0.04 |
| TLI | 0.98 | 0.94 |

**Table S3. SEM indices of goodness of fit.** CFI = Comparative Fit Index, RMSEA = Root Mean Square Error of Approximation, SRMR = Standardized Root Mean Square Residual, TLI = Tucker Lewis Index, AD = Alzheimer’s Disease.


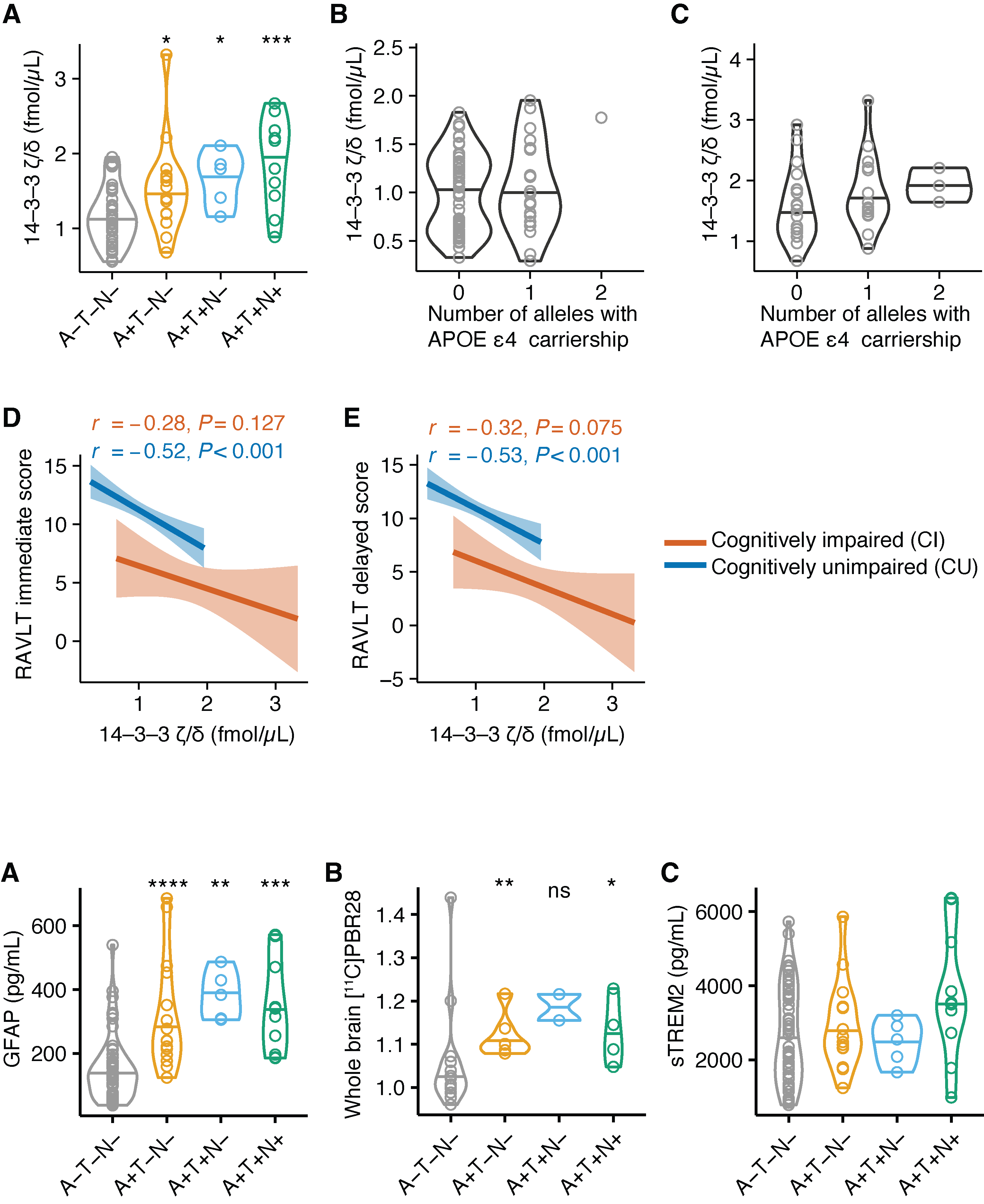


**Fig. S1. 14-3-3** $\boldsymbol{\zeta/\delta}$ **is associated with memory deficits in cognitively unimpaired. (A)** CSF levels of 14-3-3 $\zeta/\delta$ in A-T-N-, A+T-N-, A+T+N-, and A+T+N+, cognitively unimpaired young individuals were excluded for this analysis. Wilcoxon-test with FDR-correction for multiple comparisons was used for statistical comparisons. * *P*<0.05**,** *** P<0.001. **(B-C)** CSF levels of 14-3-3 $\zeta/\delta$ in CU (B) and CI (C) participants divided by APOE ε4 carriership. **(D-E)** Spearman correlation analyses between CSF levels of 14-3-3 $\zeta/\delta$ and RAVLT immediate score (D) and delayed score (E) in cognitively unimpaired and impaired participants. Exact *P*-values and correlation coefficients are shown in the figure.


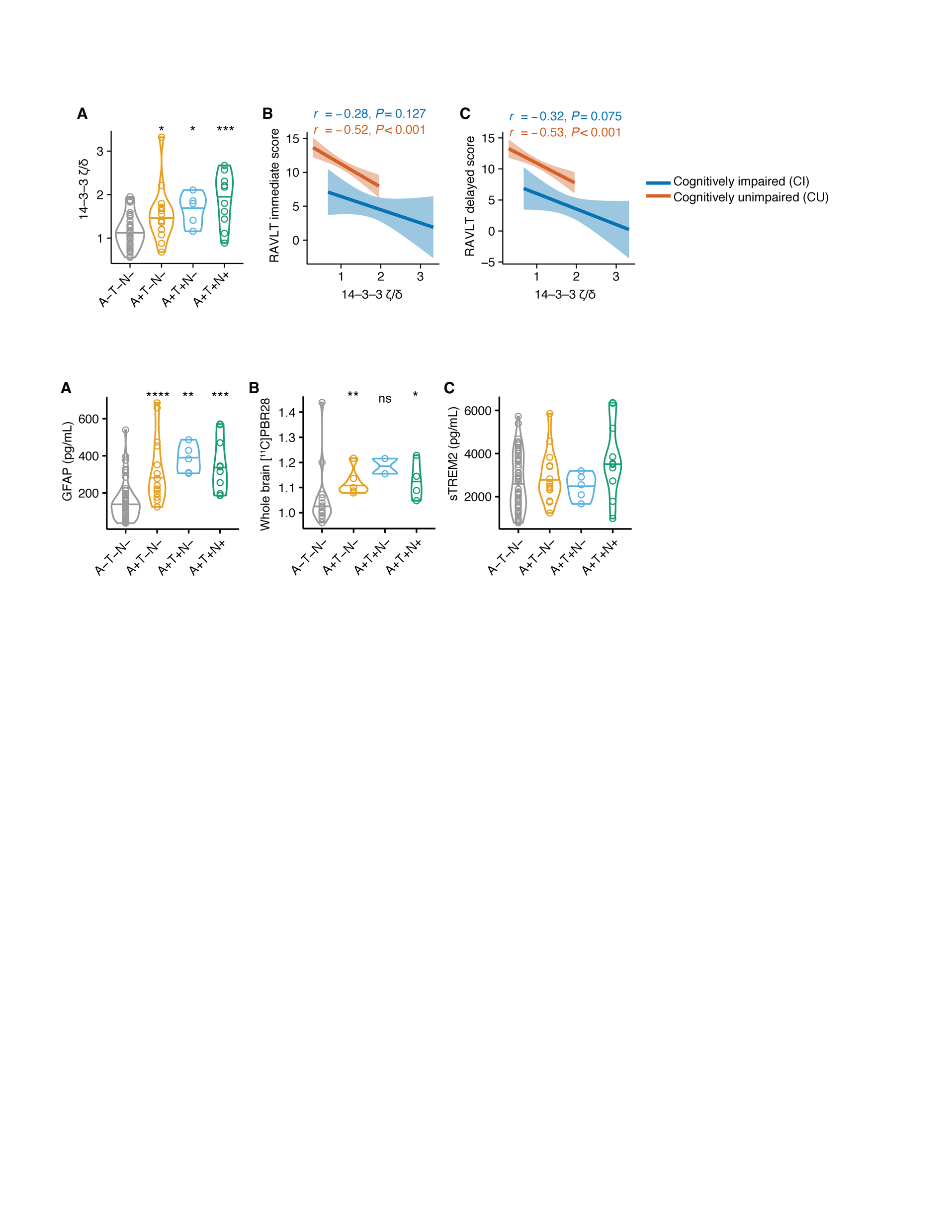


**Fig. S2. Astrogliosis and microglia activation are detectable in early disease. (A–C)** Plasma GFAP levels (A), whole brain [^11^C]PBR28 SUVR (B) and CSF sTREM2 (C) levels in A–T–N–, A+T–N–, A+T+N–, and A+T+N+ according to the A/T/N framework using [^18^F]AZD4694, [^18^F]MK6240 and hippocampal volume. Wilcoxon-test against A–T–N– with FDR-correction for multiple comparisons were conducted. **P*< 0.05, ***P*<0.01, ****P*<0.001.
